# The emerging role of norepinephrine in cognitive dysfunctions of Parkinson's disease

**DOI:** 10.3389/fnbeh.2012.00048

**Published:** 2012-07-25

**Authors:** Elena M. Vazey, Gary Aston-Jones

**Affiliations:** Laboratory of Neuromodulation and Behavior, Department of Neurosciences, Medical University of South CarolinaCharleston, SC, USA

**Keywords:** Parkinson's disease, norepinephrine, locus coeruleus, executive dysfunction

## Abstract

Parkinson's disease (PD) is the second most common neurodegenerative disorder, affecting 1% of the population over age 60. In those patients cognitive dysfunction is a persistent issue that impairs quality of life and productivity. Neuropathological studies demonstrate significant damage in brain regions outside the nigral dopamine (DA) system, including early degeneration of locus coeruleus norepinephrine (LC-NE) neurons, yet discussion of PD and treatment focus has remained dopaminergic-based. Motor symptoms benefit from DA replacement for many years, but other symptoms including several cognitive deficits continue unabated. Recent interest in non-DA substrates of PD highlights early involvement of LC-NE neurons and provides evidence for a prodromal phase, with cognitive disturbance, even in sporadic PD. We outline insights from basic research in LC-NE function to clinical and pathological evidence highlighting a role for NE in PD cognitive dysfunction. We propose that loss of LC-NE regulation, particularly in higher cortical regions, critically underlies certain cognitive dysfunctions in early PD. As a major unmet need for patients, research and use of NE drugs in PD may provide significant benefits for cognitive processing.

Canonically, Parkinson's disease (PD) is a neurodegenerative movement disorder with progressive loss of substantia nigra (SN) dopamine (DA) neurons that modulate cortical-striatal loops (Hoehn and Yahr, 1967; Litvan et al., 2003; Dickson et al., 2009). Accordingly, there is a long history of investigation and therapeutic development targeting DA replacement strategies for PD. Products of this research have dramatically improved the management of many motor symptoms for PD patients, but DA replacement has a complex interaction with cognitive symptoms (Wolters, 2009; Barone, 2010; Kehagia et al., 2010a). The relative success in addressing motor symptoms in PD has highlighted the paucity of validated treatments for non-motor symptoms, including many cognitive dysfunctions. Studies in PD subpopulations have identified a number of genetic variants associated with known susceptibility or increased risk of PD (Dickson et al., 2009). Studies of cognitive abilities in those individuals, and retrospective studies in sporadic PD, demonstrate that cognitive dysfunction can occur in pre-motor/prodromal stages of disease and is prevalent throughout disease progression, even in patients who do not develop dementia (PDD; Halliday et al., 2008; Leverenz et al., 2009; Keri et al., 2010). Cognitive dysfunction in early PD includes fronto-executive deficits with impairments in planning, working memory, attentional control, and cognitive flexibility; in contrast, PDD patients go on to develop hallucinations as well as significant deficits in verbal and visuospatial learning and memory (Kehagia et al., 2010a). Cognitive symptoms in PD have significant impact on quality of life, life expectancy, and caregiver stress (Diaz and Waters, 2009). The presence of cognitive dysfunction in PD patients treated successfully for motor symptoms indicates the need for, and emergence of, research into additional neural substrates of cognitive dysfunction in PD. Further study in genetically susceptible individuals, and in early stages of sporadic PD, is warranted to develop hypotheses on the progression of cognitive function in PD and design interventional strategies to treat unmet needs in these patients.

There are a number of non-DA neuropathological features in PD, including significant loss of locus coeruleus norepinephrine (LC-NE) and nucleus basalis of Meynert (nbM) cholinergic (ACh) neurons (Braak et al., 2003; Halliday et al., 2006). Executive functions, seated in the prefrontal cortex (PFC), rely on ascending input from several neuromodulatory systems including DA, serotonin, ACh, and prominently NE (Aston-Jones and Cohen, 2005; Chudasama and Robbins, 2006; Robbins and Arnsten, 2009). They are also regulated by SN-DA input to fronto-striatal loops (Floresco and Jentsch, 2011), the loss of which is the pathological hallmark of PD. Overt Lewy body pathology in PFC structures occurs late in PD (Hawkes et al., 2010) and may be associated with PDD, as is nbM loss (Zweig et al., 1993). In contrast, early executive dysfunctions likely result from loss of PFC neuromodulation from subcortical structures such as LC. Post-mortem analyses from PD patients and MPTP models have identified reductions in frontal NE and serotonin, but not in DA levels, highlighting potential substrates for executive dysfunction in PD (Nayyar et al., 2009; Goldstein et al., 2011). Within the CNS, Braak staging identifies a caudorostral progression of PD with Lewy pathology in LC-NE neurons occurring before that in SN-DA or ACh populations. LC-NE pathology is posited to occur more than 10 years before clinical diagnosis of PD (Braak et al., 2003; Hawkes et al., 2010). During this period prodromal/early PD symptoms include functions classically associated with LC-NE such as mood and sleep disturbances (Hawkes, 2008). We propose that the associated early executive dysfunction may be due, at least in part, to LC-NE pathology as these cognitive deficits are independently associated with LC-NE function in numerous animal studies (Berridge and Waterhouse, 2003; Aston-Jones and Cohen, 2005). LC-NE pathology/loss is thought to contribute to PD progression, occurring early and in advance of DA loss, and continuing throughout PD (Mavridis et al., 1991; Gesi et al., 2000; Srinivasan and Schmidt, 2003). Integrating cognitive studies on LC-NE function with PD pathology and animal models may identify novel insights for early diagnosis and early interventions to improve current therapeutic strategies. Below, we outline a growing body of evidence from both clinical and preclinical investigations for a potentially prominent yet underexplored role for LC-NE in cognitive dysfunction of PD, specifically in measures of cognitive inflexibility.

## Cognitive flexibility and PD-related cognitive inflexibility

Adaptive cognitive function requires updating goals or strategies to redirect attention when environmental or homeostatic conditions dictate; i.e., cognitive flexibility. Coordinating efficient behavioral execution of complex tasks by integrating goal and rule representations with control processes, memories, and attention is well established as an essential function of PFC (as reviewed in Miller and Cohen, 2001; Aston-Jones and Cohen, 2005; Robbins and Arnsten, 2009; Floresco and Jentsch, 2011). Deficits in cognitive flexibility are a DA resistant hallmark of early PD that impacts PD patients and their caregivers on a daily basis.

Lesion and imaging studies across species have strongly implicated the PFC in the flexibility of cognitive processing (Miller and Cohen, 2001; Kehagia et al., 2010b). Multiple paradigms evaluate components of cognitive flexibility, from fundamental rapid response inhibition to more complex set-shifting control (Floresco and Jentsch, 2011). Set-shifting tasks are akin to the classical Wisconsin Card Sorting Task (WCST), in which the subject must sort each card into one of three categories based on the face figures: shape, number, color. Once the subject has learned the correct rule, the rule is changed without warning and the subject must learn the new rule by trial and error. This task reveals profound deficits in early PD, as well as other disorders with PFC dysfunction (Lees and Smith, 1983; Owen et al., 1993; Konishi et al., 1999). In set-shifting tasks, compound or multi-dimensional stimuli are presented (e.g., shapes and overlaid lines), and discrimination between stimuli in one dimension is required (e.g., shapes instead of lines). After acquiring the discrimination rule, the rule changes unexpectedly requiring a shift in attentional strategy to identify the new dimension requiring attention (e.g., line direction). This is dependent on not only suppression of a previously learned rule (e.g., square shape) but also exploration of previously irrelevant stimuli or dimensions (e.g., lines) and development of a new attentional set (e.g., vertical lines denote correct stimulus). This strategy change is termed an extra-dimensional shift (EDS) because attention to one dimension of stimuli (shapes) must be abandoned and shifted to another (previously irrelevant) dimension (lines). Neuropsychological assessments using set-shifting tasks such as the WCST or CANTAB IED (intra-extra dimensional set shift) tasks are now standard clinical practice for evaluating cognitive flexibility. These evaluations have been translated into preclinical set-shifting task (SST) analogs for rodents (Birrell and Brown, 2000; Ragozzino, 2002; Floresco et al., 2008). Imaging, lesion and reversible inactivation studies across species have implicated lateral PFC in particular (rodent mPFC) in EDS (Birrell and Brown, 2000; Floresco et al., 2009). Early-stage PD patients show relatively preserved performance in reversal learning and intradimensional shifts (IDSs—changing discrimination strategies within a dimension, e.g., square to circle) but are significantly impaired in EDS (changing between dimensions, e.g., shape to line; Downes et al., 1989; Owen et al., 1992). Basic neuroscience work from several approaches indicates that NE regulation of PFC is critical to attention and cognitive flexibility including EDS in SST (Chudasama and Robbins, 2006; McGaughy et al., 2008; Braver et al., 2009). Deficits in cognitive flexibility and LC-NE pathology co-occur early in PD, and likely in prodromal phases; the similar onset of LC pathology and cognitive inflexibility in PD may indicate a link between this pathology and symptom. In animal studies, LC-NE lesions directly produce PD-like deficits in executive function, including cognitive inflexibility (Tait et al., 2007; McGaughy et al., 2008). Until recently, EDS deficits in PD had been postulated to be a consequence of DA depletion in PFC or DA mis-regulation of cortico-striatal connections (Monchi et al., 2004; Nagano-Saito et al., 2008). In PD the SN-DA neurons are affected whereas the predominant DAergic input to PFC arises from the ventral tegmental area, a population relatively spared in this disease (Hirsch et al., 1988; Braak et al., 2004; Maingay et al., 2006). Neither L-dopa nor D1 agonists restores EDS performance in PD patients, despite improving motor function and cortical fMRI activity (Lange et al., 1992; Jubault et al., 2009). This further indicates that non-DA systems are critically involved in PFC mediated flexibility deficits in PD.

## LC-NE system and cognitive flexibility

LC is the sole source of NE throughout cerebral cortex, and projects especially to parietal, temporal, and frontal lobes (Foote et al., 1983; Berridge and Waterhouse, 2003), areas prominently involved in cognitive functions. Our lab found that LC neurons in behaving animals exhibit two modes of activity, phasic (short bursts of 8–10 Hz spikes), and tonic (2–5 Hz baseline activity; Usher et al., 1999). These recording studies, in view of associated behaviors and effects of NE on target neurons, led Aston-Jones and Cohen (2005) to propose the Adaptive Gain Theory, which states that the function of the two LC activity modes is to regulate the balance between *exploitation* of known behavioral outcomes for a familiar strategy (phasic mode) vs. *exploration* of possible new outcomes with a novel strategy (e.g., flexible behavior, tonic mode), thereby maximizing behavioral utility, i.e., response-related rewards. This and several other prominent theories for LC function, including the Neural Interrupt theory (Dayan and Yu, 2006), Reorienting System theory (Corbetta et al., 2008), Network Reset theory (Bouret and Sara, 2005), and State Modulation hypothesis (Berridge and Waterhouse, 2003), all converge in their attempts to identify LC-NE output as a core element in the regulation of cognitive flexibility.

LC-NE activity can cause rapid and complex responses in cortical targets (Florin-Lechner et al., 1996; Berridge and Abercrombie, 1999; Bouret and Sara, 2004). NE release increases the gain of target cell activity, i.e., NE increases the responsiveness of target cells to other inputs (Woodward et al., 1979; Servan-Schreiber et al., 1990; Waterhouse et al., 1998). We have proposed that this gain increase, occurring for a phasic LC response when a decision has been reached, acts to boost processing in circuits engaged by the decision, increasing task-related focus (Clayton et al., 2004; Aston-Jones and Cohen, 2005). By doing this, phasic LC responses are thought to promote task-related behavioral responses (e.g., exploit known behavioral outcomes). However, when behavioral success (utility) declines, LC neurons increase tonic (baseline) activity and lose phasic, task-related responses. As described in more detail elsewhere (Aston-Jones and Cohen, 2005), this tonic LC activity facilitates disengagement from a task by temporally decoupling LC activity from task execution and increasing the responsiveness of LC target neurons to non-task related events (e.g., previously irrelevant dimensions), facilitating exploration and behavioral flexibility.

Several studies show that LC-NE function is inextricably linked to cognitive flexibility, particularly EDS performance (Tait et al., 2007; McGaughy et al., 2008). In clinical studies, EDS ability is impaired early in PD, when LC-NE neurons are lost (Chan-Palay and Asan, 1989; Petrovitch et al., 2011). Preclinically, atipamezole, an NE α2 antagonist that increases NE release, improves EDS shifting in a manner blocked by local mPFC α1 antagonists (Lapiz and Morilak, 2006). Desipramine, a NE reuptake blocker, also improves EDS performance and increases extracellular NE release in mPFC during set shifting, particularly when given chronically (Lapiz et al., 2007). Recently, the specific NE reuptake inhibitor atomoxetine (ATM) was shown to rescue EDS deficits elicited by selective lesions (via DBH saporin toxin) of LC-NE fibers in rat mPFC (Newman et al., 2008). The EDS deficits produced by these lesions concur with those produced by neurochemically specific (6-OHDA) lesions of the dorsal noradrenergic bundle (DNAB) of LC-NE projections to forebrain (Tait et al., 2007; McGaughy et al., 2008). The intersection of conclusions from behavioral neurophysiology studies in animals showing a role for LC in exploration and behavioral flexibility (described above) with these from animal studies of LC lesions strongly supports the view that LC-NE plays an important role in cognitive flexibility. We propose that when task utility declines, tonic activity increases in LC neurons, which increases gain (synaptic responsivity) throughout the CNS at widespread LC targets. This tonic and widespread gain increase facilitates activity in non-task-related circuits and thereby augments transitions among representations for other tasks or rules (i.e., increases exploratory behavior and cognitive flexibility). In PD, we hypothesize that degeneration of LC-NE neurons dampens the NE-mediated modulation needed to disrupt the ongoing task and augment competing circuits, in effect inhibiting task disengagement and flexible behavior and promoting perseverative responding.

## LC-NE as a therapeutic target for PD

NE modulation is a viable therapeutic target in PD due to the proven safety and efficacy of NE modulators in other disorders, early involvement of LC in PD neuropathology, opportunity for concurrent efficacy in other non-motor symptoms (depression, sleep disorders) as well as potential impact on disease progression (as reviewed by Fornai et al., 2007; Rommelfanger and Weinshenker, 2007). A number of small trials have shown improvements in DA replacement therapy side effects and non-motor symptoms with therapies that target NE (Carpentier et al., 1996; Bedard et al., 1998; Rascol et al., 2001; Pintor et al., 2006; Jankovic, 2009). In relation to cognitive function only a few small trials directly investigating NE replacement have been undertaken in PD patients. The specific NE reuptake inhibitor ATM led to improvements in clinical global impression scales of cognition in two recent studies (Marsh et al., 2009; Weintraub et al., 2010). Cognitive flexibility was not examined in those studies, but in preclinical reports ATM improved EDS deficits produced by specific LC-NE lesions in PFC (Newman et al., 2008). Evidence of pathology in the LC-NE system *in vivo* and preclinical studies described above identify unanswered questions on a possible role for LC-NE neuromodulation in early PD cognitive dysfunction. Can the preclinical role of LC-NE in behavioral flexibility translate into a viable therapeutic target for PD? Only future studies of NE modulation on measures of cognitive flexibility in PD patients can answer this, and such studies would provide valuable additions to our understanding of PD pathology and the role of NE in cognitive processing.

## LC-NE interactions with DA and ACh in PD cognitive inflexibility

PD pathology encompasses many systems that contribute to various cognitive impairments. Widespread DA loss is the fundamental pathology of PD, but evidence for a role of striatal DA in cognitive flexibility is inconclusive (Collins et al., 2000; Jubault et al., 2009); this may be due to compensatory mechanisms in PFC following loss of striatal DA regulation. 6-OHDA lesions of marmoset PFC led to cortical-subcortical DA imbalance and impaired initial task acquisition, but did not impair reversal or EDS performance; in fact, by disrupting maintenance of the attentional set these lesions appeared to improve EDS (Roberts et al., 1994; Crofts et al., 2001). Local antagonism of D1 or D2 transmission in mPFC causes mild impairments in set-shifting, but local infusions of D1 or D2 agonists in mPFC of rodents do not affect SST (Floresco et al., 2006). These findings are similar to clinical studies where DA-based treatments for PD do not improve cognitive flexibility but may be more important in other PD related executive impairments such as planning and spatial working memory (Jubault et al., 2009; Kehagia et al., 2010a).

Of note in relation to cognitive dysfunction in PD is nbM degeneration. Because LC-NE innervation activates nbM ACh neurons, LC-NE pathology early in PD may contribute to decreased cortical ACh levels and subsequent cholinergic dysfunction in PD (Jones, 2004; Calabresi et al., 2006; Espana and Berridge, 2006). Ascending progression of Lewy body pathology with degeneration in nbM ACh neurons occurs in many PD patients, and is more pronounced with PDD, the severity may be a contributing factor in the development of PDD (Bohnen et al., 2003; Hilker et al., 2005; Jellinger, 2006). Concurrent use of cholinergic inhibitors also contributes to cognitive decline and development of dementia in PD patients, whereas cholinesterase inhibitors can provide significant benefits for patients with PDD (Ehrt et al., 2010; Kehagia et al., 2010a). Performance on semantic fluency tests (dependent on posterior cortical integrity as opposed to PFC functions) are also strong predictors of progression to PDD, demonstrating a separation of NE- and ACh-dependent aspects of cognitive processing in early PD vs. PDD, respectively (Williams-Gray et al., 2007). Evidence for cholinergic involvement specifically in cognitive flexibility as part of the early prodromal dysexecutive phenotype in PD is limited; in fact, ACh denervation has been shown to have no effect on EDS performance (Robbins and Roberts, 2007; McGaughy et al., 2008). It appears ACh has a significant role in cognitive processing in PD, but that it is more pertinent to later stages of PD with the development of PDD; also, cholinergic function in PD may be influenced by loss of ascending LC-NE input.

## Focus on the locus: loss of LC-NE neurons and cognitive impairment in early PD

Evidence is increasing for an important role of LC in multiple neurodegenerative disorders, including PD (German et al., 1992; Gesi et al., 2000; Weinshenker, 2008). We and others have argued that LC has a number of functions besides its roles in cognitive processing, including regulation of sleep, mood, and neuroinflammation, all of which are perturbed in PD and may benefit from NE-based therapies (Aston-Jones and Bloom, 1981; Aston-Jones et al., 1996; Gonzalez and Aston-Jones, 2008; Heneka et al., 2010). Until now, LC loss in PD has primarily been investigated for contributing to nigral DA neuron death, exacerbating motor dysfunction and dyskinesias (Srinivasan and Schmidt, 2003; Fulceri et al., 2007; Rommelfanger et al., 2007). In individuals with a genetic predisposition to PD, cognitive flexibility deficits have been identified up to 5 years prior to onset of motor symptoms (Keri et al., 2010). In idiopathic PD, cognitive dysfunction is commonly present below Hoehn and Yahr Stage 2 and prevails throughout disease progression, even in advanced patients who do not develop full dementia (Downes et al., 1989; Green et al., 2002; Rodriguez-Ferreiro et al., 2010). Very little has been done to incorporate basic research on LC-NE in cognition with the corresponding cognitive impairment and neuropathology in PD. As discussed above, several animal studies find that depleted NE in PFC results in poor EDS performance, and that this impairment is resolved by manipulations that increase NE transmission (Tait et al., 2007; Newman et al., 2008).

## Concluding perspective

PD is characteristically a dopaminergic disorder but DA resistant non-motor symptoms, including dysexecutive features appear early if not in prodromal phases of PD. As prefrontal DA levels are largely preserved throughout PD, other systems are likely substrates. It is postulated that LC-NE pathology also begins early in PD progression. Involvement of LC-NE system has been recognized as part of PD neuropathology with Lewy body accumulation, reductions prefrontal NE levels, and loss of LC neurons. The role LC-NE pathology plays in PD, particularly in cognitive processing, is underexplored and only beginning to emerge. We propose that loss of prefrontal NE input may contribute to executive dysfunction and specifically EDS deficits early in PD. We know from extensive preclinical investigation that prefrontal LC-NE projections have a critical role in executive function, particularly mechanisms underlying EDS. Currently there are gaps between our understanding of LC-NE function and application of NE targeted therapies in PD research and treatment. Future studies directly investigating the potential role of LC-NE in executive function in PD could lead to novel therapies and insight into LC-NE in cognitive processing and pathological processes in PD.

### Conflict of interest statement

The authors declare that the research was conducted in the absence of any commercial or financial relationships that could be construed as a potential conflict of interest.
